# Secure mobile agents for efficient medical information retrieval: A verifiable variable threshold secret sharing approach

**DOI:** 10.1371/journal.pone.0325950

**Published:** 2025-06-20

**Authors:** Pradeep Kumar, Sur Singh Rawat, Kakoli Banerjee, Ayodeji Olalekan Salau, Gyanendra Kumar, Niraj Singhal

**Affiliations:** 1 Department of Computer Science and Engineering, JSS Academy of Technical Education, Noida, Uttar Pradesh, India; 2 Department of Electrical/Electronics and Computer Engineering, Afe Babalola University, Ado-Ekiti, Nigeria; 3 Saveetha School of Engineering, Saveetha Institute of Medical and Technical Sciences, Chennai, Tamil Nadu, India; 4 Chitkara University Institute of Engineering and Technology, Chitkara University, Rajpura, Punjab, India; 5 Department of IoT and Intelligent Systems, Manipal University Jaipur, Jaipur, Rajasthan, India; 6 Sir Chhotu Ram Institute of Engineering and Technology, Chaudhary Charan Singh University, Meerut, Uttar Pradesh, India; Beijing Technology and Business University, CHINA

## Abstract

Mobile Agents are a new type of computing that is replacing the client-server approach. Mobile agents are little pieces of code that function automatically on behalf of the owner. Many applications, such as e-commerce, parallel computing, network management, and health care, use mobile agents. The healthcare industry is one of the most growing fields in any country. As the population increases day by day the requirement of medical resources is proportionally increasing. Due to high patient demand and a severe lack of medical resources, a remote medical healthcare system is required. However, the deployment of remote healthcare systems over the Internet introduces a new set of challenges, including interoperability among heterogeneous networks and the need to navigate through multiple public systems dispersed over insecure networks. This paper explores how mobile agents can effectively tackle these challenges, especially in heterogeneous and potentially malicious environments. A key focus of this research is the development of a mathematical model for secure medical information retrieval. This model incorporates a variable threshold secret-sharing mechanism, employing the Chinese remainder theorem and multiplicative inverse with modular arithmetic at different levels. By integrating these cryptographic techniques, the proposed approach ensures the confidentiality and integrity of medical information during its retrieval, contributing to the overall safety and robustness of mobile agent computing in healthcare scenarios.

## 1. Introduction

Medical facilities represent one of the most crucial concerns in human civilizations, as they directly impact on the quality of residents’ lives. Medical care is characterized by highly specialized healthcare institutions and clinical divisions [1]. The healthcare industry is not only dispersed and fragmented but also diverse, exhibiting significant regional autonomy. Due to the sheer volume of medical data, coupled with its complexity and diversity, traditional computation paradigms falter when attempting to simulate such an environment.

**Fig 1:**
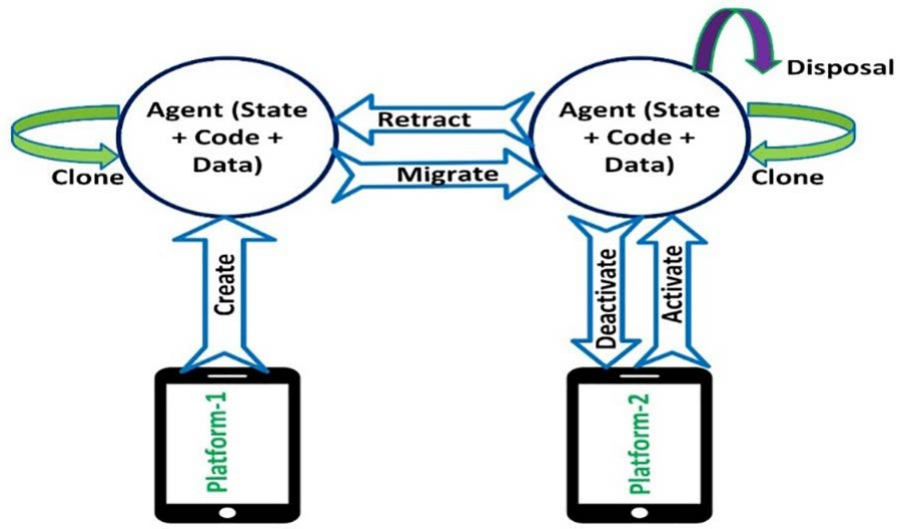
Mobile Agent life cycle.

In this context, we delve into the effective utilization of the mobile agent paradigm within the healthcare domain. Mobile agents consist of small programs that migrate as distinct independent units from one platform to another. They can execute processes on a platform, suspend their execution, migrate to a different platform, and resume their execution there. Mobile agents [2] adhere to a specific lifecycle for executing assigned tasks, as depicted in Fig 1.

Given the inherently self-driven mobility of mobile agents across distributed networks, they often interact with malicious agents and platforms.

In the present era, one of the burgeoning paradigms for overseeing applications in distributed computing is the Mobile Agent. Various client-server paradigms, such as Remote Procedure Call (RPC), Message Passing Interface (MPI), and code on demand, were utilized in distributed computing prior to the advent of mobile agent technology. Numerous benefits have been introduced in the computing landscape, including the automatic dynamic execution of tasks, reduced channel load, and minimized latency.

A mobile agent [3] moves seamlessly across a network, transitioning from one machine to another. Ensuring the security of both the mobile agent and the platform it interacts with presents a substantial challenge when embracing the mobile agent paradigm.

### 1.1. Characteristic of mobile agents

1) Mobility: Agents can temporarily halt their execution on one agent platform and then resume it on another, namely, in a different location. This phenomenon is commonly known as migration.
2) Self-determination: Each mobile agent operates based on a code specifically crafted to accomplish one or more objectives. The actions performed by mobile agents are solely determined by this code, without any direct interaction from external entities.
3) Reactivity: Agents adapt to environmental changes to accomplish their objectives.
4) Proactively: Agents alter their surroundings and employ a variety of actions to attain their objectives.
5) Sociability: This pertains to an agent’s capacity to engage in communication with other agents. Given that certain agents rely on communication with others to observe their environment, this attribute is of paramount importance.

**Fig 2:**
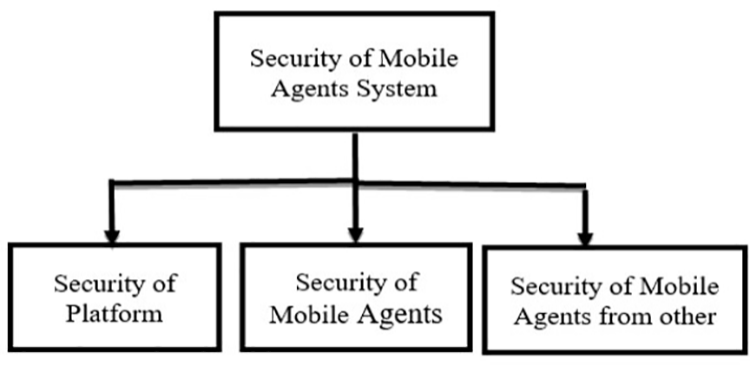
Security of mobile agents.

Mobile agent Security as shown in Fig 2 is categorized into three main parts: Security of Mobile agent, Security of Platform, and Security of mobile agent Network.

### 1.2. Mobile agent applications in the healthcare domain

The utilization of the Mobile Agent paradigm is widespread and applied in numerous real-life applications, such as electronic commerce, networking, and personal assistance. Employing mobile agent technology entails the collaboration of multiple agents: each agent is assigned specific responsibilities, which collectively break down the entire task.

Healthcare operates within a vast open ecosystem [4] characterized by decentralized decision-making and care management. This necessitates the exchange of complex and diverse data types among various clinics. During emergencies, there arises a need to collaborate on patient information from different medical institutes to review the patient’s medical history. Mobile agent technology stands as one of the most popular and sought-after strategies for accessing patient medical history information [5]. Through cooperation and coordination of their actions, mobile agents present a natural approach to resolving distributed problems with heterogeneous inputs. They also proactively carry out tasks that can be beneficial to the user. Within the medical domain, various applications of mobile agents abound.

1) Medical data management: Providing information from different medical data sources and consolidating it.
2) Decision-making support: Approaches converged to aid healthcare practitioners in completing tasks such as treatments and diagnostics.
3) Securing medical information: Methodologies for enhancing the security and privacy of patient data.
4) Patient management: Medical information acquisition, analysis, and protection systems are critical to providing high-quality patient care.
5) Medical training and education: Mobile technology is used in medical training and education.
6) Remote care or telemedicine: Patients’ status can be monitored remotely, allowing for more comprehensive care.
7) Planning and resource allocation: Used in planning and resource allocation.
8) Information retrieval and integration: Methodologies for retrieving medical data from disparate databases.

**Fig 3:**
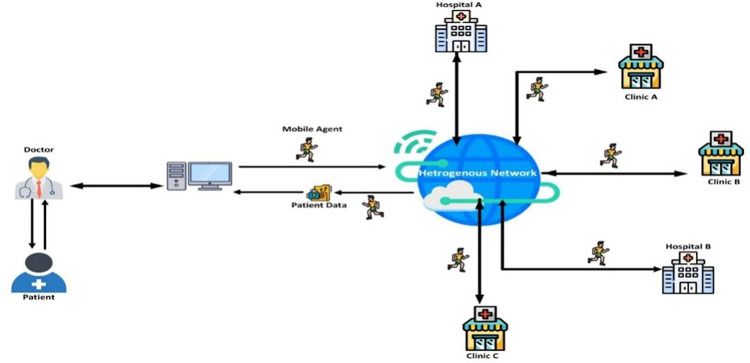
Mobile Agents application in the medical field.

Medical services are perhaps the most pressing issue in human social order, as the existing nature of humans directly relies upon healthcare services. Medical services are data-driven and information-driven. The application of mobile agents in the medical field is shown in Fig 3.

Healthcare in the medical sector is one of the most intricate undertakings due to the absence of all medical facilities in a centralized location. The healthcare industry is not only dispersed and fragmented, but it also exhibits considerable diversity, along with significant local autonomy. Furthermore, within the medical sector, patient information holds paramount importance, and a majority of treatments are critical. Given the vast volume of information, as well as the complexity and diversity of data, conventional computational methods prove inadequate in functioning effectively.

Mobile agent computing [6] plays a pivotal role in addressing challenges within the healthcare sector. Mobile agent programming has emerged as a prominent standard for organizing web-based applications, wherein these agents migrate across a network to execute tasks beyond their host machines. Each task is executed independently by the mobile agent. Mobile agents find extensive applications in the healthcare domain, encompassing decision-making systems, accessing distributed data sources, and coordinating healthcare-related tasks. This is due to their adaptive, proactive, and cooperative nature.

The principal objective of this article is to ensure the security of mobile agents during migration, as these agents carry sensitive patient personal data and medical reports. The proposed framework is founded on the multilevel Chinese remainder theorem, incorporating a variable threshold value to safeguard patient data and medical reports.

### 1.3. Contribution of the paper

The contribution of this paper is summarized as follows:

1. This paper presents a novel approach to bolster the security of mobile agents and platforms by employing multilevel key management and dynamic threshold values.
2. A dynamic threshold value is essential for both the mobile agent and platform, catering to diverse levels of authentication requirements. To secure the migration of mobile agents effectively, we propose a technique grounded in the Multilevel Chinese Remainder Theorem.
3. For the execution and authentication of mobile agent migration, we generate multilevel secret keys, each assigned a dynamic threshold value specific to its corresponding level.

### 1.4. Organization of the paper

The article is organized as follows: In Section 2, we primarily delve into related work. Our proposed approach, along with preliminaries, is detailed in Section 3. The performance evaluation and implementation of our approach are addressed in Section 4. Section 5 concludes the article, highlighting the key findings and avenues for future work.

## 2. Related work

Mobile agent technology has been explored in numerous fields, including the medical sector, to enhance secure and efficient data processing, resource management, and task execution. This work delves into key concepts and potentially related work associated with the deployment of secure mobile agents within the medical field.

Srivastava *et al.* [7] introduced a technique aimed at enhancing the security of mobile agents. Their approach ensures the confidentiality of these agents in both the communication channel and the execution environment, while also preserving their integrity. To safeguard mobile agents from hostile execution environments, it becomes imperative to limit the security verification authority on the execution environment side. This involves adopting an agent-driven methodology wherein the mobile agent safeguards its sensitive code and data from the external malicious environment. The author proposed a novel technique for a critical component of mobile agents. To guarantee confidentiality, a private key is generated, composed of two key parts: one generated through a hash function and the other generated randomly (AES-generated).

The authors in [8] present a significant contribution in their work by introducing the concept of Trust Score, an original metric for assessing platform reliability. They introduce a Trust Score-based Itinerary Arranging Algorithm designed to assist Mobile Agents in making dynamic decisions informed by the Trust Score. To enhance the Trust Scoring system, they introduce the Trustability Coefficient of Variation. Although the Trust Scoring system generates separate sections for a server platform, the framework consolidates these sections using the Coefficient of Variation, culminating in a unified metric referred to as the Trustability Coefficient of Variation. This approach leverages the Trustability Coefficient of Variation to rank server platforms according to experimental results, showcasing its superiority over existing alternative support networks.

Geetha *et al.* [9] presented a dual encryption mechanism aimed at safeguarding data blocks, effectively addressing concerns such as snooping and alteration. This approach guarantees the confidentiality, integrity, authentication, authorization, and scalability of Mobile Agents (MA). The proposed model ensures the security of free-roaming MAs through Trustworthy Roaming Model and cryptographic algorithms, providing resilience against both passive and active attacks, specifically connived truncation attacks.

Han *et al.* [10] addressed the public key management challenges in mobile networks by proposing the “Trust Delegation” concept based on an ID-based cryptosystem, which enhances security. This concept enables multiple applications concurrently while remaining resilient against fundamental transparency and key loss. Their design, which minimizes transaction quantity and complex elements like Home Subscriber Server and Bootstrapping Function, proves to be robust against denial of service (DoS) attacks targeting HSS or BSF.

Raji *et al.* [11] proposed a distinctive approach ensuring anonymity for both agent owners and itineraries. By utilizing a public anonymizer system consisting of auxiliary hosts, the owner employs this system for each step of the agent’s journey. These hosts serve as intermediaries, maintaining security. Security remains an emerging area within MA systems, and substantial work remains to be accomplished.

Cavalcante *et al.* [12] Stated the benefits of agent technology for application systems with advanced attributes like autonomy and cooperative problem-solving. They emphasize the necessity of ensuring the security of such agent-based ecosystems, and many security approaches have been proposed by researchers to meet security requirements.

The authors in [13] presented a secret-sharing technique based on the Chinese Remainder Theorem (CRT), termed multilevel threshold secret sharing (MTSS). Their scheme divides share into levels, each with a distinct threshold value for secret key recovery. Only when a specific number of valid shares are available at each level can the secret be reconstructed. Xingxing Jia *et al.* [14] proposed a novel TCSS (Threshold Secret Sharing) framework based on the CRT, offering reduced share size and recovery complexity compared to previous schemes.

Kandar *et al.* [15] presented a verified secret-sharing approach based on shadow sharing to safeguard the reconstruction of secret information. Their system also includes a strategy for detecting cheaters, easing updates for the combiner and dealer, and protecting against various security risks.

Bagga *et al.* [16] discuss the use of Mobile Agents for organizing, processing, and retrieving medical data from heterogeneous sources, aiding healthcare decisions. Agents assist healthcare providers during treatment and enhance the security of health data stored in local repositories. Chen *et al.* [17] considered multiple public systems distributed across unsecured heterogeneous networks, addressing these challenges with the use of Mobile Agents. They propose a safe access control mechanism based on the Chinese Remainder Theorem and discrete logarithm to ensure secure patient data monitoring and control. M. van der Haak *et al*. [18] aim to identify legal requirements for data security and data protection in cross-institutional electronic patient records (EPR), exploring methods to achieve these requirements.

Jung *et al.* [19] highlight the application potential of agent technology in systems with advanced qualities like autonomy and dynamic problem-solving abilities. They emphasize the need for security within such agent-based ecosystems and discuss various security approaches proposed by researchers.

Ruxandra *et al.* [20] focused on Verifiable Secret Sharing (VSS), ensuring consistent secret reconstruction even if a malicious dealer distributes invalid shares. Verma *et al.* [21] proposed a hybrid-based VSS scheme for communicating secrets in a multilevel setting, aiming to create effective and secure systems. Zhao *et al.* [22] presented a verifiable multi-secret sharing (VMSS) scheme based on the YCH scheme and the intractability of the discrete logarithm, enhancing verification while reducing computation quantity. Das *et al.* [23] introduced a one-way collision-resistant hash function-based multi-secret sharing approach with general access structures, offering flexibility and reduced computational costs. B. Orgun *et al.*[24] Discussed a framework based on the multi-agents and ontology to provide better communication in distributed health care system without considering the limitation of the client server approach. The aurthour introduced the Electronic Medical Agent System (eMAGS), a multi-agent system utilizing an ontology based on the Health Level Seven (HL7) public health messaging standard. The aim is to streamline the flow of patient data across the healthcare sector. In a separate work, Su [25] detail the design and implementation of a mobile multi-agent platform-based open information system (IMAIS). This system incorporates an automated diagnosis engine for enhanced and distributed ubiquitous fetal monitoring. The adoption of a FIPA2000 standard-compliant agent development platform, the Java Agent Development Environment (JADE), effectively addresses issues related to interoperability, scalability, and openness in diverse e-health contexts. IMAIS, when used with lightweight, portable fetal monitors, allows for continuous long-term monitoring without disrupting a patient’s daily activities or limiting mobility. The versatile system architecture is applicable to various monitoring scenarios, including elder care and vital sign monitoring.

The current body of literature addressing the security of migrating agents outlines diverse methods, yet none offers a comprehensive framework that seamlessly integrates compatible techniques into a unified security model [26,27]. Despite the advantages of employing mobile agent technology in remote healthcare, it introduces security concerns. As a mobile agent conducts its operations, traversing the Internet and engaging with various hosts and agents for information exchange, it becomes vulnerable to security threats [28]. However, the focus is gradually shifting towards developing solutions aimed at securing migrating (mobile) agents, a significantly more intricate challenge. The issue of devising a mechanism is difficult due to the independence and adaptability of mobile agents. Despite the practical benefits of Mobile Agent technology, both the mobile agent and its platform remain susceptible to various security threats [29].

## 3. Proposed solution

Security of mobile agent in the field of healthcare for securing accessing data of patient proposed multilevel secure key distribution using multilevel Chinese remainder theorem. Some preliminaries are important for proposed framework.

### 3.1. Preliminaries

In this section, we define MTSS, the CRT, Mignotte’s, and Asmuth–Bloom schemes based on the CRT, as well as other preliminaries that are crucial to our approach.

1) Multilevel threshold secret sharing:

In a multilevel threshold secret scheme, authorized set

$Let\;k_{1\;},k_{2\;},k_{3\;},\;k_{4\;}\ldots \ldots \ldots \ldots \ldots \;k_{m\;}represent\;the\;shares$, while$U_{1\;},U_{2\;},\;\;U_{3}\ldots \ldots .U_{n\;\;}the\;security\;levels.$


$${U=\{U}_{1\;},U_{2\;\;},\;\;U_{3}\ldots \ldots \ldots ..U_{n}\}={⋃}_{j=1}^{m}k_{j}$$

Let ${T=\{t}_{1\;},t_{2\;\;},\;\;t_{3}\ldots \ldots \ldots ..t_{m}\}$ be the threshold values.


$$where\;1\leq t_{j\;\;}\left|k_{1}\right|+\left|k_{2}\right|+\left|k_{3}\right|\ldots \ldots \ldots \ldots \left|k_{j}\right|,$$

$$\;for\;j=1\;to\;m.t_{1\;}<t_{2\;\;}<t_{3}\ldots \ldots \ldots ..<t_{m\;\;}$$

Authorized set of share in(k,T)multilevel scheme defined as

$$=\{\;B\subseteq \left(U_{1\;},U_{2\;\;},\;U_{3}.U_{n\;\;}\right)for\;all\;i\epsilon \left\{1,2,3\ldots \ldots \ldots m\right\}\;\&\;\left|B\cap U_{j=1}^{i}k_{j}\right|\geq t_{j}$$

where $B=\left\{U_{i1\;},U_{i2\;\;},\;\;U_{i3}\ldots \ldots \ldots ..{Ui}_{t\;\;}\right\}\&{Ui}_{k\;\;}\;\neq \;{Ui}_{kj\;\;}for\;k\neq j\;,\;any\;subset\;\{k_{1\;},\;\;i_{3\;},\;i_{4\;}\ldots \ldots \ldots \ldots \ldots \;i_{t}\;\;\;of\;\left\{1,2\ldots \ldots \ldots n\right\},$

2) Mignotte series: Mignotte series is a collection of positive integers ${\;q}_{1}<q_{2}<q_{3\ldots \ldots \ldots \ldots \ldots \ldots \ldots ..}<q_{n}$, in increasing order in such a way $q_{n-t+2}*q_{1}*\;q*q_{n}<q_{1}*q_{2}*q_{3}\ldots \ldots \ldots \ldots *q_{t}$. All positive integers’ pair wise is co-prime$.\gcd\left(q_{i\;},q_{j}\right)=1\;in\;such\;a\;way\;i\neq j.$

$${\;\;\;\;\;\;\;\;\;\;\pi }_{i=0\;\;\;qn-i<}^{t-2}\pi_{i=1\;qi\;}^{t}$$ (1)

3) Chinese remainder theorem: Chinese remainder theorem provides us with a method to uniquely determine a number x modulo t-many relatively prime integers ${\;q}_{1}<q_{2}<q_{3\ldots \ldots \ldots \ldots \ldots \ldots \ldots ..}<q_{n}$ given that:

$$x\equiv \alpha_{1}\left(modq_{1}\right)$$

$$x\equiv \alpha_{2}\left(modq_{2}\right)$$

$$\;\;\;\;\;\;\;\;\;\;\;x\equiv \;\;\;\alpha \left(modq_{3}\right)$$ (2)

$$x\equiv \alpha_{t}\left(modq_{t}\right)$$

One solution of given congruence relation


$$\;\;\;\;\;\;\;\;\;\;\;\;\;\;\;x=\;\;\;\;\;\;\;\;\;\sum_{\;\;\;i=1}^{t}\frac{Q}{q_{i}}y_{i\;}\alpha_{i}modQ$$ (3)

$$where\;\;\;\;\;\;\;Q=q_{1}*q_{2\;}*q_{3}*q_{4}\ldots \ldots \ldots \ldots \ldots q_{t}$$

### 3.2. Proposed model

In this paper, we propose a key management [27] approach for secure mobile agent migration based on a variable threshold multilevel framework utilizing the Chinese remainder theorem. A secret key is generated for authenticating both mobile agents and platforms, and it is utilized within the mobile host computer during execution. For a malicious platform or attacker attempting to access the agent’s security key, a fixed threshold value is necessary.

**Fig 4:**
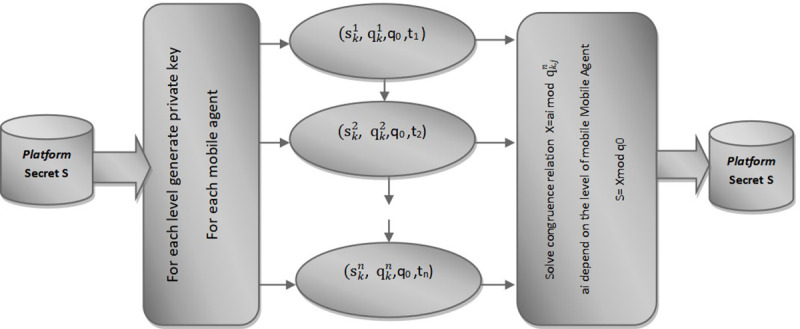
Variable Threshold model for Mobile Agent Migration (VTMMA).

The selected secret ‘S’ is randomly divided into ‘k’ partial shares through the application of the Chinese remainder theorem, as depicted in Fig 4.

The framework comprises ‘n’ levels, ranging from higher to lower levels. Higher-level mobile agent shares can be employed to reconstruct the secret for the execution of mobile agents on lower-level platforms. However, lower-level shares cannot be used to retrieve the key at upper levels. Each level of mobile agent necessitates a distinct threshold number of shares to reconstruct the key, and these threshold values vary across different levels. This differentiation in threshold values across mobile agent levels ensures higher security for the execution of mobile agents at varying levels.

The proposed framework is based on the three main phase initializations (algorithm 1), Secret hiding (algorithm 2), Share creation (algorithm 3), and reconstruction of share (algorithm 4).

**Algorithm 1** In the initialization phase, every host selects $q_{0}$ for each level. Each level has a fixed number of mobile agents and respectively pairwise coprime integers. This number selection is based on the Mignotte sequence. Hosts choose a secret key S in set of $Z_{q_{0}}$.

**Initialization**:

**Input:** Number of levels *n,* where each level has mobile agents, and integer *t* (threshold).

**Output**: Secret key S, and pairwise coprime integers ${\;q}_{0\;,}q_{1}{,q}_{2},q_{3}\ldots \ldots \ldots \ldots q_{n}$.

**Step 1: Parameter initialization**

 i. Select $q_{0}$ a random integer from $Z_{q_{0}}$ for secret key

 ii. (0 ≤ S < *q*_0_) for each level secret key.

 iii. Initialize an empty list Q to store integers $q_{0\;,}q_{1}{,q}_{2},q_{3}\ldots \ldots \ldots \ldots q_{n}$.

**Step 2: Choose pairwise coprime integers** $q_{0\;,}q_{1}{,q}_{2},q_{3}\ldots \ldots \ldots \ldots q_{n}$

*for i=1 to n*

 i. Select $q_{i}$ in such a way $q_{i}>q_{i-1}$.

 ii. $\gcd\left(q_{i},q_{j}\right)=1\;for\;every\;i\neq j\leq n.$

 iii. Append $q_{i}$in to list Q.

**Step 3: Check the Mignotte sequence condition**

 i. If $\prod_{i=1}^{t}q_{i}>(q_{0}+1).\prod_{i=1}^{t-1}q_{n-t+i+1}$

 ii. {

 iii. proceed with $q_{0\;,}q_{1}{,q}_{2},q_{3}\ldots \ldots \ldots \ldots q_{n}$

 iv. **}**

 v. **Else**

 vi. Recalculate q values and repeat from Step 2 until the condition is satisfied.

**Step 4:** Return the secret key *S* and the sequence of integers *Q.*

**Algorithm 2** Consider finite field $F_{p}$where *p* is the prime number. Select *m* any random number except 1 used for hiding the Secret ′S′. Since 1 is a multiplicative identity and cannot be used in an algorithm for hiding the secret, it is avoided. After multiplying *m* by *S*, different numbers were obtained.

**Input:-** S: Secret (integer). - *p*: Prime number (defining the finite field *F*_*p*_).

**Output:-** y: Hidden parameter. - **m**: Random number used for hiding the secret.

**Step 1: Select a random number m**

 i. Generate a random number m such that: - m ∈Zp (i.e., 1 < m < p). - m ≠ 1 (since 1 is a multiplicative identity and cannot hide the secret).

**Step 2: Compute the hidden value y**

 i. Multiply m by the secret S.

 ii. Compute y ≡ (m * S) mod p to keep the result within the finite field F_p_

**Step 3: Return the result**

 i. Return (y, m), where: – y is the hidden value. - m is the random number used to hide the secret.

**Algorithm 3** Shares are created for each level, from higher level to lower level.

**Share creation:**

**Input:**

 **-** S: Secret.

 - $q_{0\;,}q_{1}{,q}_{2},q_{3}\ldots \ldots \ldots \ldots q_{n}$: Sequence of pairwise coprime integers (for levels).

 - t: Threshold (number of shares required to reconstruct the secret).

 - p_i_: Public moduli for each mobile agent at each level (from q_0_ to q_n_).

**Output: - S**_**i**_: Public shares for each mobile agent.

**Step 1: Calculate value of α**

 **i.** $\alpha \in (\frac{\prod_{i=1}^{t}(qn-i+1)}{q0},\;\prod_{i=1}^{t}\frac{q_{i}}{q_{0}}-1)$

**Step 2: Share creation for each level (from upper to lower)**

 **i.** $S_{i}=(S+\alpha q_{0}mod\;p_{i}$ public share of every Mobile agent *0 ≤ i ≤ n*

 **ii.** *Save the public share S*_*i*_ *for mobile agent i.*

**Step 3:** ***Return the shares***

 *i.* Return the list of shares *S*_*0*_*, S*_*1*_*,..., S*_*n*_.

**Algorithm 4** At the reconstruction of the secret key for the execution of the mobile agent every upper-level mobile agent utilized lower-level share. The threshold value of each level is different in such a way, $t_{1\;}<t_{2\;\;}<t_{3}\ldots \ldots \ldots ..<t_{n\;\;}$.

**Reconstruction of share**

**Input:**

 **-** *i:* The level at which the mobile agent wants to reconstruct the secret.

 - *t*_*i*_: The threshold value for the i^th^ level $t_{1\;}<t_{2\;\;}<t_{3}\ldots \ldots \ldots ..<t_{n\;\;}$

 - q_0_,: Pairwise coprime moduli for respective mobile agents.

 - $s_{k}^{i}$ The shares from the lower-level mobile agents.

 - *α*_*j*_: The value α_j_ used for share creation at level j.

**Output: – S: Reconstructed secret key.**

**Step 1: Initialize variables for reconstruction**

 i. Mobile agent wants to reconstruct share at *i*^*th*^ level

 ii. i = i^th^ level/*choose level */

 iii. t_i_ = threshold value of each mobile agent to regenerate secret

 iv. $t_{1\;}<t_{2\;\;}<t_{3}\ldots \ldots \ldots ..<t_{n\;\;}$

**Step 2: Use shares from the lower levels to reconstruct the share at level i**

 i. Each level of a mobile agent has a different threshold as compared to other levels that provide higher security to mobile agent execution at different levels.

 ii. $q_{t\;j}^{j}<q_{k,\;j}^{i}<q_{nj-tj+2}^{j}$

 iii. ${(s\;+\;\alpha_{j}q_{0}\;–\;s}_{k}^{i})\;mod\;q_{k,\;j}^{i}{=\delta s}_{k,j}^{i}$ /*Public parameter ${=(\delta s}_{k,j}^{i},\;q_{k,\;j}^{j})$ in such a way *i < j* with respective mobile agent.

 iv.a_i_ depend on the level of mobile Agent

**Step 3: Solve the system of congruences to reconstruct the secret**

 i.Solve the system of congruence relations using the Chinese Remainder Theorem (CRT): $X\equiv a_{i}\;mod\;{\;q}_{k,j}^{n}$

**Step 4: Final reconstruction of the secret**

 i. $S\equiv \;X\;mod\;q_{0}$

**Algorithm 5** Generated secret authentication based on the algorithm verification of secret key. if *(y = m.S′modq*_*0*_) satisfies the relationship, the generated key is correct otherwise is wrong.

**Verification of secret key**

**Input:**

 - y: Public parameter.

 - m: Random number used for hiding the secret Key.

 - q_0_: Modulus prime number with in finite field.

 - x: Some value that, when reduced modulo q_0_, gives the candidate secret S′.

**Output: –** Message indicating whether the generated secret is correct or wrong.

**Step 1: Compute secret key** *S′*

 *i. Calculate S′* ≡ *xmodq*_*0*_

 ii. Step 2 Verify the relationship

 *iii. If (y = m.S′modq*_*0*_)

 iv. {

 v. “Generated secret is Correct”.

 vi. else

 vii. “Generated secret is wrong”.

#### 3.2.1. Example of proposed model.

The mathematical example below serves as an illustration of our suggested strategy.

The host chooses a secret key for authentication of mobile agents at the time of execution, S = 102, and q_0_ = 113, at level one in the subset, L_1_, the integers associated with shareholders, ${\;U}_{1}^{k}$, *k* = 1, 2, 3, 4,…… are ${\;q}_{1}^{1}$ = 137, ${\;q}_{2}^{1}$ = 139, and ${\;q}_{3}^{1}$ = 250. The t_1_-threshold range is (250, 19043). The dealer selects α_1_ = 150 and *s + α*_*1*_ *q*_*0*_ = 17052 which is in the above range. The shares are $s_{1}^{1}$= 64, $s_{2}^{1}$ = 94, and $s_{3}^{1}$ = 52

In the subset, L_2_, the integers associated with shareholders, ${\;U}_{2}^{k}$, *k* = 1, 2, 3, 4, are ${\;q}_{1}^{2}$ = 293,${\;q}_{2}^{2}$= 307, ${\;q}_{3}^{2}$= 313, and ${\;q}_{4}^{2}$ = 319. The t_2_-threshold range is (99847, 28154663). The dealer selects α_2_ = 6864 and *s + α*_*2*_*q*_*0*_ = 775 734 which is in the above range. The shares are $s_{1}^{2}$ = 163, $s_{1}^{2}$ = 252, $s_{3}^{2}$ = 120 and $s_{4}^{2}$ = 245.

In the subset, L_3_, the integers associated with shareholders, ${\;U}_{3}^{k}$, *k* = 1, 2,..., 7, are ${\;q}_{1}^{3}$ = 229, ${\;q}_{2}^{3}$= 233, ${\;q}_{3}^{3}$ = 239, ${\;q}_{4}^{3}$ = 241, ${\;q}_{5}^{3}$ = 277, ${\;q}_{6}^{3}$ = 281, and ${\;q}_{7}^{3}$ = 283. The t_3_-threshold range is (22027871, 3073309843). The dealer selects α_3_ = 194 946 and s + α3q_0_ = 22029000 which is in the above range. The shares are $s_{1}^{3}$ = 116, $s_{2}^{3}$ = 15, $s_{3}^{3}$ = 131, $s_{4}^{3}$ = 154, $s_{5}^{3}$ = 21, $s_{6}^{3}$ = 5, and $s_{7}^{3}$ = 280.

For generation of secret key in higher level 3, used ${\;U}_{1}^{3}$ from subset L_1_, ${\;U}_{3}^{2}\;and\;{\;U}_{4}^{2}$ from subset L_2_. Mobile agents choose moduli ${\;q}_{3,3}^{1}$, ${\;q}_{3,3}^{2}$, ${\;q}_{4,3}^{2}$ are 263,269 and 251 respectively using condition ${\;q}_{t,j}^{j}$*<*${\;q}_{k,j}^{i}$*<*${\;q}_{nj-tj+2}^{j}$. Where j is higher level and i is lower-level j > i.241=${\;q}_{4}^{3}$<${\;q}_{1,3}^{1}$,$q_{1,3}^{2},{\;q}_{4,3}^{12}$*<*${\;q}_{5}^{3}$ = 277. Calculate public parameter associated to ${\;U}_{k}^{i}$.

Mobile agent evaluates *(s + α*_*3*_*q*_*0*_*-*$s_{3}^{1}$*)mod*${\;q}_{3,3}^{1}$.

(22029000-52) mod263 = 68 then public information of ${\;U}_{3}^{1}$ in subset *L*_*3*_is (68,263). Similarly, the public parameter ${\;U}_{3}^{2}$ and ${\;U}_{4}^{2}$ are (201,269) and (242,251) respectively.

**Case 1:** Assume that at level 3 for generation of secret key collaboration of ${\;U}_{3}^{1}$ from *L*_*1*_, ${\;U}_{4}^{2}$ from *L*_*2*_, ${\;U}_{3}^{3}$ and $U_{6}^{3}$ from *L*_*3*_ are required. Four shares are required to regenerate the secret key level 3 because at level three threshold is ‘t_3_=4’.

$s_{3}^{1}$ = 52, $s_{4}^{2}$ = 245, $s_{3}^{3}$ = 131 and $s_{6}^{3}$ = 5. Public information of (68,263), (242,251) ${\;q}_{3}^{3}$ = 239, and ${\;q}_{6}^{3}$ = 281 are used to compute secret key.


$$x=(s_{3}^{1}+{\delta s}_{3,3}^{1})\;mod\;{\;q}_{3,3}^{1}$$ (4)

$${x=(s}_{4}^{2}+\;{\delta s}_{4,3}^{12})\;mod\;{\;q}_{4,3}^{2}$$ (5)

$$x=\left(s_{3}^{3}\right)mod\;{\;q}_{3}^{3}$$ (6)

$${x=(s}_{6}^{3})\;mod{\;q}_{6}^{3}$$ (7)

x=(52+68) mod 263

x=(245+242) mod 251

x=131 mod 239

x=5 mod 281

after apply CRT to solve congruent relation x = 22029000. Secret share is reconstructed as s = x modq_0_, 22029000%113 = **102.**

**Verification of Secret:** public parameter are (y,m) =(63,97) If y = S*m% q_0_

102*97%113=63 correct. Generate secret is correct.

**Case 2:** Assume that at level 3 for generation of secret key collaboration of ${\;U}_{3}^{2}$and ${\;U}_{4}^{2}$from L_2_ level, *and* ${\;U}_{1}^{3}$ *and* ${\;U}_{2}^{3}$from L_3_, are required. Number of partial shares at level three are 4. To find the secret key by solving the system of equations as follows


$${x=(s}_{3}^{2}+{\delta s}_{3,3}^{2})\;mod{\;q}_{3,3}^{2}$$ (8)

$${x=(s}_{4}^{2}+{\delta s}_{4,3}^{2})\;mod)\;{\;q}_{4,3}^{2}$$ (9)

$${x=(s}_{1}^{3})\;mod)\;{\;q}_{1}^{3}$$ (10)

$$x=(s_{2}^{3})\;mod)\;{\;q}_{2}^{3}$$ (11)

x=(120+201) mod 269

x=(245+242) mod 251

x=116 mod 229

x=15 mod 233

After apply CRT to solve congruent relation x = 22029000. Secret share is reconstructed as s = x modq_0_22029000%113 = **102**

**Verification of Secret:** public parameter are (y,m) =(63,97), If y = S*m% q_0_

(102*97)%113=63 correct. Generate secret is correct.

**Case 3:** Assume that at level 3 for generation of secret key collaboration of ${\;U}_{3}^{2}$ from L_2_ level,${\;U}_{4}^{2}$from L_3_, and ${\;U}_{5}^{3}$ from level L_3_are required. Number of partial shares at level three is but the shod value for regeneration of key is 4. So, can’t not generate the key by using three partial shares,


$$x=\left(s_{3}^{1}+{\delta s}_{3,3}^{1}\right)\;mod\;{\;q}_{3,3}^{1}$$ (12)

$${x=(s}_{4}^{2}+{\delta s}_{4,3}^{2})\;mod\;{\;q}_{4,3}^{2}$$ (13)

$$x=\left(s_{5}^{3}\right)mod\;{\;q}_{5}^{3}$$ (14)

x= (52+68) mod 263

x= (245+242) mod 251

x = 21mod277, after apply CRT to solve congruent relation x = 3743399. Secret share is reconstructed as s’ = x’ modq_0_3743399%113 = **48** which is not equal of secret key.

**Verification of Secret:** public parameter is (y, m) = (63, 97). If y = S*m% q_0_, (48*97) %113 = 23 not equal to 63 correct. Generate secret is incorrect.

## 4. Results and discussion

This section discusses the significance of the findings in the healthcare industry after presenting the outcomes of using secure mobile agents in conjunction with the Verifiable Variable Threshold Secret Sharing (VVTSS) approach for effective medical information retrieval [30].

### 4.1. Security measurements and implementations

Table 1 presents the comparison of proposed approach with other methodology previously proposed on the basis of different parameter Validity (V), Traceable (T), Confidentiality (C) and Consistency (C). As a result, the proposed approach if far better than the other.

**Table 1:** Comparison by using security parameter.

S. No	Scheme Name	Year	V	C	C	T
1	A new efficient (t, n) verifiable multi-secret sharing (VMSS) based on YCH scheme	2005	yes	yes	No	yes
2	A practical verifiable multi-secret sharing scheme	2007	yes	yes	No	yes
3	An efficient threshold verifiable multi-secret sharing	2008	yes	yes	No	yes
4	An efficient multi-use multi-secret sharing scheme based on hash function	2010	yes	yes	No	yes
5	Multilevel threshold secret sharing based on the Chinese remainder theorem	2014	yes	yes	No	yes
6	Efficient verifiable multi-secret sharing scheme based on hash function	2014	yes	yes	Yes	yes
7	Dealer-leakage resilient verifiable secret sharing	2014	yes	yes	No	yes
8	A Hybrid-Based Verifiable Secret Sharing Scheme Using Chinese Remainder Theorem	2019	yes	yes	yes	yes
9	Proposed (VTSSS)	–	yes	yes	yes	Yes

Table 2 presents the comparison of proposed approach on the basis of parameter Proactive, Threshold, Verifiable, single/multiple authentications and Change Secret. As a result, the proposed approach is far better than the other.

**Table 2:** Comparison of Different Technique on the basis of different parameter.

S. No	Name of Author	Year	Technique Used	Proactive	Threshold	Verifiable	Single/Multiple	Change Secret	Change Access Scheme Structure
1	Blakley	1979	Vector space based	0	1	0	Single	–	Easy to add new share
2	Shamir	1979	Polynomial based	0	1	0	Single	–	Easy to add new share
3	Benaloach	1980	Circuit based	0	0	0	Single	–	–
4	Asmuth and Bloom	1983	CRT Based	0	1	0	Single/Multiple	–	–
5	Mignotte	1986	CRT Based	0	1	0	Single	–	–
6	In gemarrssonm and Simmous	1991	Linear Block Based	0	1	0	Single	Easy	Easy to add new share
7	Franklin and yung	1992	Polynomial based	0	1	0	Multiple	Easy	–
8	Pederson	1992	Polynomial based	0	1	1	Single	–	Easy to add new share
9	Brickell	1995	Vector space based	0	0	0	Single	–	–
10	He and Dawson	1995	Polynomial based	1	1	0	Multiple	Easy	Easy to add new share
11	Hezberg	1995	Polynomial based	1	1	0	Single		Easy to add new share
12	Noar and Shamir	1995	Visual Secret sharing	0	1	0	Single	–	–
13	Ghodosi	1998	Polynomial based	0	0	0	Single	–	–
14	Bai	2006	Matrix Projection based	1	1	0	Multiple	Easy	Easy to add new share
15	Iftene	2007	CRT Based	0	0	0	Single	–	
16	Feldman	2008	Polynomial based	0	1	1	Single	–	Easy to add new share
17	Pang	2008	Polynomial based	1	1	1	Multiple	Easy	
18	Lein Harn	2014	CRT Based	1	1	0	Multiple	–	Easy to add new share
19	Om Prakash Verma	2020	CRT Based and Polynomial	1	1	1	Multiple	–	Easy to add new share
20	Proposed (VTSSS)		Proposed algorithm CRT and Multiplicative inverse	1	1	1	Multiple	–	Easy to add new share

The proposed approach is based on the fusion of the multiplicative inverse concept and the Chinese remainder theorem with a variable threshold value. It is compared with three other approaches: the Reputation-based Model (RBM), Trust Scoring System (TSS), and Trust Ranking System (TRS). These three approaches provide only a single level of authentication and lack verification. Table 3 presents a comparison of the proposed approach with the three techniques based on the number of mobile agents and the time taken for authentication key generation [31].

**Table 3:** Variable Threshold secret sharing based Multilevel Chinese remainder theorem implementation.

No of mobile agent	Level 1, t = 2				Level 2, t = 3				Level 3, t = 4			
	Selected share	Selected Modulo	Original Share	Generated Share	Selected share	Selected Modulo	Original Share	Generated Share	Selected share	Selected Modulo	Original Share	Generated Share
**5**	(10, 16)	(13, 19)	244	244	(4, 8, 11)	(13, 11, 19)	30	30	(7, 12, 17, 18)	(11, 17, 19, 23)	7777	7777
**10**	(4, 38)	(11, 41)	202	202	(15, 5, 1)	(17, 11, 29)	610	610	(12, 4, 1, 5)	(31, 23, 11, 13)	694	694
**15**	(7, 24)	(47, 31)	148	148	(26, 9, 10)	(53, 19, 17)	503	503	(42, 4, 14, 17)	(43, 31, 19, 37)	128	128
**20**	(53, 13)	(59, 31)	230	230	(12, 58, 5)	(47, 71, 13)	200	200	(5, 0, 29, 51)	(53, 31, 47, 83)	217	217
**25**	(31, 2)	(83, 17)	529	529	(22, 3, 0)	(83, 37, 47)	188	188	(9, 71, 54, 42)	(59, 73, 103, 107)	363	363
**30**	(85, 95)	(89, 113)	886	886	(20, 8, 52)	(29, 59, 83)	716	716	(7, 29, 88, 56)	(23, 83, 89, 97)	444	444
**35**	(58, 37)	(131, 67)	975	975	(0, 25, 44)	(43, 107, 73)	774	774	(20, 19, 9, 21)	(41, 73, 23, 131)	676	676
**40**	(23, 1)	(43, 23	668	668	(14, 3, 93)	(139, 41, 149)	987	987	(22, 19, 24, 97)	(47, 23, 67, 173)	962	962
**45**	(90, 103)	(227, 179)	998	998	(54, 45, 84)	(179, 47, 149)	233	233	(1, 16, 68, 14)	(23, 89, 131, 149)	461	461
**50**	(60, 60)	(197, 103)	60	60	(173, 36, 6)	(191, 53, 19)	937	937	(75, 17, 8, 16)	(193, 29, 67, 59)	75	75
**55**	(88, 88)	(131, 89)	88	88	(34, 81, 4)	(41, 199, 23)	280	280	(54, 78, 3, 15)	(181, 173, 11, 97)	597	597
**60**	(4, 171)	(167, 199)	171	171	(65, 2, 85)	(107, 13, 103)	600	600	(220, 18, 71, 14)	(263, 31, 103, 67)	483	483
**65**	(65, 26)	(251, 29)	172	172	(0, 10, 3)	(11, 29, 31	561	561	(3, 39, 17, 116)	(223, 211, 131, 139)	672	672
**70**	(172, 172)	(251, 227)	328	328	(31, 53, 13)	(59, 107, 127)	267	267	(43, 240, 107, 300)	(67, 337, 269, 307)	914	914
**75**	(74, 27)	(127, 43)	262	262	(193, 6, 1)	(401, 23, 71)	995	995	(261, 95, 8, 160)	(353, 109, 137, 269)	967	967
**80**	(100, 100)	(139, 313)	100	100	(20, 149, 97)	(193, 257, 103)	406	406	(8, 92, 17, 229)	(13, 137, 53, 313)	229	229

In this table, as the number of mobile agents in a malicious environment increase, the time required for key generation also increases proportionally. The RBM, TSS, and TRS do not rely on the threshold value, whereas the proposed approach is based on variable threshold values at multiple levels for generating the secret key used in authentication [32].

In proposed framework implement a variable threshold based secret sharing model for mobile agent security. Every mobile agent utilize multiple level ach level has a unique key for execution of assigned task. And each level has different threshold to generate the secret key. In our implementation considering three level and level one, level two and level three consider threshold 2, 3, 4 respectively. Secret share distributed among the mobile agents. Reconstruction of secret key is allowed only when required minimum number of mobile agent collaborated. Implementation of different mobile agents for secure accessing of data there is requirement of secret key. Here consider three levels each level required fixed number of secret shares of mobile agents to regenerate the key to accessing the resources of platform. Higher level utilizes lower level of share to regenerate the share but vice versa not possible. Table 3 show the all calculation regarding implementation of proposed framework.

In Table 4, ‘16’ epochs are used for different values of the number of mobile agents [33]. Upon observation, it is noted that the proposed approach’s authentication processing time is significantly better than that of the RBM and TSS, but it takes longer compared to the TRS. However, the TRS approach employs single-level authentication, resulting in the proposed approach being the superior choice among the three. In order to create a mobile agent using a typical Python toolkit, we can use a number of frameworks and modules, such as Pykka which enable agent-based modelling, mobility, and interaction across distributed settings. With Pykka, you can use the actor-based model to simulate mobile agents that have the ability to move and interact. It’s perfect for simulating locally [34]. To simulate and analyze the results for the algorithm that secures mobile agents for medical data retrieval using the VVTSS approach, enhanced by a Multilevel CRT, the following steps can be taken:

**Table 4:** Comparison of response time among the Reputation-based Model, Trust Scoring System, and Trust Ranking System and VTSSS.

S. No	No of Mobile Agents	Response time in seconds			
		RBM Single level (sec)	TSS Single level (sec)	TRS Single level (Sec)	VTSSS Multiple level (sec)
1	5	0.48	0.36	0.18	0.000445
2	10	0.93	0.69	0.34	0.000429
3	15	1.39	1.05	0.53	0.009259
4	20	1.94	1.41	0.71	0.000404
5	25	2.28	1.82	0.9	0.000384
6	30	2.85	2.15	1.1	0.017359
7	35	3.44	2.48	1.2	0.000367
8	40	3.84	2.85	1.4	0.000369
9	45	4.35	3.25	1.6	0.000381
10	50	4.76	3.54	1.74	0.000375
11	55	5.22	3.89	1.95	0.000365
12	60	5.96	4.33	2.18	0.023819
13	65	6.25	4.65	2.34	0.000404
14	70	6.68	5.05	2.5	0.00691
15	75	7.14	5.38	2.69	0.000462
16	80	7.67	5.73	2.85	0.00673

**Fig 5:**
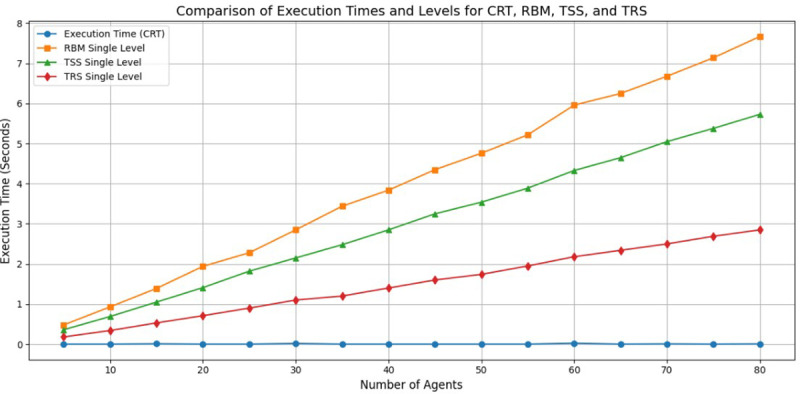
Response time comparison for different trust models as the number of mobile agents.

Fig 5 shows the response time comparison for different trust models as the number of mobile agent’s increases:

- **RBM:** Response time increases the most significantly as the number of mobile agents grows.
- **TSS:** Has moderate response times, higher than TRS but lower than RBM.
- **TRS:** Shows the lowest response time across all agent numbers.
- **VTSSS:** Multiple level system response times are lower than RBM and TSS but slightly higher than TRS, especially as the number of agent’s increases.

This simulation provides a way to secure mobile agents in medical data retrieval using a verifiable variable threshold secret sharing approach, enhanced by the Multilevel CRT. The analysis helps measure the time efficiency, security strength, and verification effectiveness.

The Verifiable Variable Threshold Secret Sharing (VVTS) Approach using Multilevel CRT can be added to the preceding table and graphs in order to compare it with the current scenarios. Usually, the VVTS method with multilevel CRT offers effective key reconstruction, scalability, and a high degree of security.

### 4.2. Comparison parameters

Many factors, including time complexity, computational overhead, and communication cost, security level, and scalability, can be assessed in order to conduct a thorough analysis of secured mobile agents across multiple metrics and scenarios [35].

**Fig 6:**
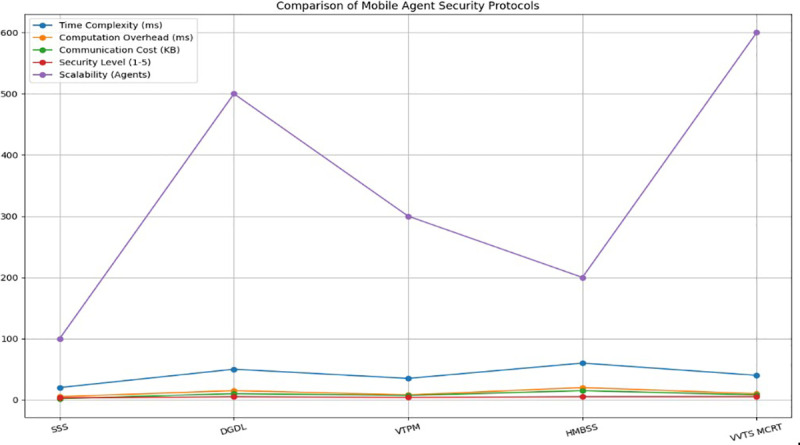
Comparison of different algorithm with proposed framework.

Fig 6 show the Comparison table of mobile agents across multiple metrics and scenarios.

- **Time Complexity:** Multiple levels of verification and modulus computations may result in moderate to high time complexity for VVTS employing multilayer CRT.
- **Computational Overhead:** The multilevel key reconstruction and verification phases make it slightly more complex than simple methods like Shamir’s.
- **Communication Cost:** Moderate, since some data transmission is required for key distribution and verification.
- **Security Level:** The threshold key generation and verification process is safe and effective thanks to CRT.
- **Scalability:** High, because the CRT can handle various security levels and secret reconstruction with ease.

Table 5 presents the comparison table of mobile agents across multiple metrics and scenarios.

**Table 5:** Comparison table of mobile agents across multiple metrics and scenarios.

S. No.	Security Protocol using for Mobile Agent	Time Complexity (ms)	Computation Overhead (ms)	Communication Cost (KB)	Security Level (1–5)	Scalability (Agents)
1	Shamir’s Secret Sharing (SSS)	20	5	2	3	100
2	Double Generalized Discrete Logarithm (DGDL)	50	15	10	5	500
3	Vectorized Tree Parity Machine (VTPM)	35	8	7	4	300
4	Hilbert Matrix-Based Secret Sharing (HMBSS)	60	20	15	5	200
5	VVTS with Multilevel CRT (VVTS MCRT)	40	10	8	5	600

Table 6 illustrates the comparison of the proposed model, which relies on multilevel secret sharing, with similar techniques. The parameters for comparison include secret generation time, secret distribution time, reconstruction time, scalability, and threshold value.

**Table 6:** The performance comparison with other Secret sharing schemes.

Scheme	Secret Generation	Share Distribution	Reconstruction time	Scalability	Threshold (*t*)
Shamir Secret share	O(twhere t= threshold number of share	O(n)	$O\left(k^{2}\right)worst\;case$	Less Scalable	Fixed threshold
Polynomial based	O(twhere t= threshold number of share	O(n)	$O\left(k^{2}\right)worst\;case$	Less Scalable	Fixed threshold
Multilevel *CRT Based*	O(n)	O(n)	$O\left(n^{2}\right)worst\;case$	High Scalable	Each level used different threshold

After analyzing these various parameters, it becomes evident that secret key sharing based on the multilevel security using the Chinese Remainder Theorem offers distinct advantages over other techniques. The Multilevel security approach is based on CRT secret sharing exhibits greater efficiency in terms of share size when compared to Shamir’s Secret Sharing. This efficiency is attributed to the employment of modular arithmetic in CRT, enabling the achievement of smaller shares. This reduction in share size contributes to minimizing storage requirements and lowering transmission overhead.

## 5. Conclusion and future scope

This paper suggested a secure framework for mobile agents that use VVTSS to retrieve medical information efficiently. The crucial problem of protecting privacy and security in the flow of sensitive medical data across dispersed contexts is addressed by this framework. The Multilevel CRT in conjunction with the VVTS technique greatly improves the security and effectiveness of medical data retrieval in mobile agent systems. The comparative analysis shows that VVTS maintains a high security level of 5 and supports scalability for up to 600 agents while achieving a time complexity of 40 ms, a computational overhead of 10 ms, and a communication cost of 8 KB. Traditional techniques, like Shamir’s Secret Sharing, on the other hand, have a time complexity of 20 ms, a lower security level of 3, and less scalability—they can only handle up to 100 agents. In addition to fixing the flaws in the current models, the VVTS framework performs better than them on important performance indicators. Potential future research directions include expanding the VVTS framework’s capability beyond 600 agents, improving its scalability for even bigger networks, and investigating how to integrate it with block chain technology for improved data integrity and traceability. Furthermore, to confirm the VVTS approach’s efficacy, flexibility, and usefulness and to guarantee its resilience to new security risks in mobile agent environments, real-world deployments in healthcare settings can be carried out.
